# Integrating Dialectical Behaviour Therapy Into the Treatment of Adolescent Depression: A Retrospective Study

**DOI:** 10.62641/aep.v53i4.1963

**Published:** 2025-08-05

**Authors:** Caiqin Xi, Xiaomei Jiang, Yanyan He, Yinping Liu, Huahua An, Keyi Shang, Xiaojing Ma, Dong Ren

**Affiliations:** ^1^The First Clinical Medical College of Gansu University of Chinese Medicine, 730000 Lanzhou, Gansu, China; ^2^Department of Psychosomatic and Sleep Medicine, Lanzhou Petrochemical General Hospital (The Fourth Affiliated Hospital of Gansu University of Chinese Medicine), 730060 Lanzhou, Gansu, China

**Keywords:** adolescent, depression, dialectical behaviour therapy, sertraline

## Abstract

**Background:**

Globally, the prevalence of depression among adolescents is on the rise, posing serious societal problems. Dialectical behaviour therapy (DBT) and selective serotonin reuptake inhibitors (SSRIs), such as sertraline, are two commonly employed therapeutic approaches that have shown good clinical outcomes. This study aims to investigate the therapeutic effects of DBT with or without sertraline on adolescent depression.

**Methods:**

This retrospective analysis reviewed 88 cases of adolescent depression treated at our hospital and compared them with 60 healthy adolescents. The patients with depression were divided into three groups: sertraline alone, DBT alone, and combined DBT and sertraline (DBT+sertraline) treatment. In the Sertraline-only and DBT+sertraline groups, sertraline was administered orally for a continuous period of 24 weeks. In the DBT-only and the DBT+sertraline groups, DBT treatment lasted for 13 weeks, followed by an observation period of another 11 weeks. DBT treatment efficacy was evaluated using the Hamilton Depression Rating Scale (HAMD) and the Cognitive Emotion Regulation Questionnaire (CERQ) at baseline and after 5, 9, 13 and 24 weeks of treatment.

**Results:**

Results showed that all three treatment modalities significantly reduced HAMD scores (*p* < 0.001, η = 0.749). The combined treatment group achieved the fastest reduction in HAMD score at the initial treatment stage. Whilst the Sertraline group showed a pronounced reduction by Week 13, it later exhibited a rebound in scores at 24 weeks, unlike the DBT-containing groups. In terms of emotional regulation strategies, CERQ scores indicated that DBT+sertraline significantly increased positive emotional regulation strategy scores, followed by DBT alone (DBT+sertraline vs DBT, *p* < 0.001), whilst the sertraline-alone group had the smallest increase (DBT+sertraline vs sertraline, *p* < 0.001) This pattern was particularly pronounced in the Positive Reappraisal subscale. Negative emotional regulation strategy scores were significantly reduced across all treatment groups, especially for the Self-blame item, with the largest reduction observed in the DBT+sertraline group, followed by the DBT alone (DBT+sertraline vs DBT, no significance) and sertraline-alone (DBT+sertraline vs sertraline, *p < *0.001) groups.

**Conclusions:**

This study's findings demonstrate that DBT and sertraline can improve emotional regulation abilities and effectively alleviate symptoms of depression in adolescents. In particular, superior outcomes were observed in the combined treatment group compared to the individual treatment groups. These findings aim to provide guidance and reference for clinicians, mental health professionals, policymakers and families of patients.

## Introduction

Depression, also known as depressive disorder, can be triggered by various factors and is a severe mental health issue that leads to persistent feelings of sadness and a loss of interest in activities [1, 2]. Adolescent depression significantly impacts the mental health of patients and can profoundly affect their academic performance, social skills and future quality of life. Moreover, depression increases the risk of heart disease, hypertension, diabetes, disability and even suicide [3, 4]. Globally, 34% of adolescents aged 10–19 years are at risk of clinical depression, surpassing the reported estimates for 18–25-year-olds; furthermore, the proportion of adolescents with severe depression reaches 8% [5]. These statistics are increasing worldwide. In the United States, the prevalence of adolescent depression rose from 8.1% in 2009 to 15.8% in 2019, nearly doubling over a decade [6]. In China, the incidence of adolescent depression has reached 22.2% [7]. The prevalence of depression is increasing annually, and the age of onset is decreasing, progressively impacting the health of children and adolescents [8]. According to the World Health Organisation’s (WHO) global burden of disease assessment report, depression is the second highest burden and disabling disease amongst all diseases by 2020 and is projected to be the largest burden of disease in the world by 2030 [9]. These statistics underscore the seriousness of adolescent depression as a global public health issue and highlight the need for effective intervention measures by the international community.

The causes of adolescent depression include personal factors and environmental influences. Personal factors encompass genetics, personality traits and physiological influences [10]. Environmental factors include family environment issues (e.g., strained family relationships, parental divorce and domestic violence) and school pressures (including academic stress and bullying). Depression can lead to risks, such as suicide, eating disorders, attention deficit hyperactivity disorder (ADHD) and poor academic performance, amongst other serious mental health issues [11].

The course of depression is long; thus, it requires prolonged, standardized treatment that may impose heavy economic and emotional burdens on families. Compared to adult depression, the course of depression in children and adolescents is longer, in which the younger the onset age, the higher the recurrence rate and the risk of suicide, severely affecting patients’ academics, family relationships and social interactions [12]. At the same time, the complexity of depressive disorders necessitates a multidimensional treatment strategy, including pharmacotherapy, psychotherapy, and social support [12]. In treating adolescent depression, pharmacological treatments commonly involve selective serotonin reuptake inhibitors (SSRIs), such as Fluoxetine and sertraline, which work by adjusting neurotransmitters in the brain to improve mood and alleviate depressive symptoms [13]. However, pharmacotherapy can cause side effects, such as nausea and fatigue [14].

Psychotherapies like dialectical behaviour therapy (DBT) and interpersonal therapy (IPT) help adolescents by identifying and changing unhealthy thought and behaviour patterns, or by improving interpersonal skills, thereby helping them establish healthier coping strategies [12, 15]. DBT is a cognitive-behavioural based psychotherapeutic approach that emphasises finding a balance between ‘change’ and ‘acceptance’, helps patients learn to cope with emotional distress and improves interpersonal relationships through individual therapy, group skills training and telephone counselling [16]. Additionally, family therapy considers the impact of one’s family environment and interactions, aiding treatment by optimising these factors. In clinical practice, a combination of medication and psychotherapy is often used to enhance treatment effectiveness [17].

Despite extensive research on these treatment methods and their applications, numerous challenges remain in clinical practice, including treatment accessibility, acceptance by patients and families and how to tailor personalised treatment plans in accordance with the specific needs of patients [18]. Furthermore, for adolescents, a special group, the treatment process must also consider their developmental stages and individual differences.

Treatment can begin with either single psychotherapy or antidepressant therapy, and if a single medication is ineffective, combined treatment can be considered [19]. Some studies have explored the differences in treatment outcomes between individual psychotherapy or pharmacotherapy and combined treatment in treating adult depression or anxiety disorders. For instance, a meta-analysis of a randomised clinical trial comparing the effects of antidepressant medication, psychotherapy and combined treatment showed that combined treatment has a statistical advantage in clinical settings, especially in severe depression, panic disorder and obsessive–compulsive disorder; it also appears to be more effective than using antidepressant medication or psychotherapy alone [20]. However, uncertainties remain regarding the optimal form of combining psychotherapy with antidepressant medication to maximise long-term benefits [19]. Even fewer studies have compared the effects of psychotherapy, pharmacotherapy, or combined treatment in adolescent patients with depressive disorders.

Therefore, the current study aims to build on prior research by specifically investigating the therapeutic effects of DBT and pharmacotherapy in adolescents with depression, thus contributing novel findings to this area of research. In particular, we retrospectively analysed the effectiveness of sertraline, DBT and combined DBT and sertraline treatment plans for adolescent depressive disorders in our hospital’s clinical practice. The use of tools, such as the Hamilton Depression Scale (HAMD) and Cognitive Emotion Regulation Questionnaire (CERQ) enable a more precise assessment of treatment effects, providing a scientific basis for managing and treating adolescent depression.

## Methods

### Patient Data

This study employed a retrospective analysis to collect data from adolescents who received treatment for depression at Lanzhou Petrochemical General Hospital from January 2023 to December 2023 to assess the effects of three treatment plans. In addition, 60 healthy adolescents were recruited from the same city where the hospital was located to serve as the control group. Recruitment involved the distribution of informational flyers and proceeded upon voluntary expression of interest, which was subsequently followed by obtaining parental consent and adolescent assent. These adolescents underwent a screening process to ensure that they did not currently exhibit depressive symptoms, had no history of seeking treatment for depressive disorders and met other general health criteria. These healthy adolescents were primarily employed in the study for baseline comparison.

### Inclusion and Exclusion Criteria

Inclusion Criteria: (1) diagnosis of adolescent depressive disorder in accordance with the Diagnostic and Statistical Manual of Mental Disorders, Fifth Edition (DSM-5) or the International Classification of Diseases, Tenth Revision (ICD-10) criteria [21]; (2) age between 12 and 18 years; (3) underwent one of the treatment plans for at least 6 months; (4) conscious, stable and able to communicate effectively; (5) has complete records of HAMD and CERQ scores; and (6) provided consent to participate in the treatment signed by patients or guardians. For the healthy adolescents included as controls, they must have good general physical health and no history of chronic or acute medical conditions that could significantly affect mood, cognitive function or ability to participate in the study (e.g., neurological disorders, endocrine disorders and autoimmune diseases).

Exclusion Criteria: (1) presence of severe suicide risk or recent suicidal behaviour; (2) coexisting severe physical illness, endocrine or neurological diseases; (3) currently using medications that could affect treatment outcomes or cause interactions; (4) lack of reliable follow-up data, operationally defined as the absence of the HAMD and CERQ scores at the critical 24-week study endpoint, or missing data for these measures at two or more of the other scheduled post-baseline assessment points (i.e., Weeks 5, 9 and 13), thereby precluding a comprehensive longitudinal analysis of treatment effects for that patient; and (5) not completing the anticipated minimum of 6 months of treatment.

### Treatment Plans

Pharmacotherapy (sertraline-only group): Directed by a psychiatrist, initiating medication treatment and regularly tracking efficacy and side effects. Sertraline hydrochloride (NMPA approval number: H20051076) was provided by Jingxin Pharmaceutical, Zhejiang, China. The initial dosage was 25 mg per day orally, which was then adjusted based on changes in the patient’s condition by up to a maximum of 200 mg per day, continuously administered for 24 weeks. After 24 weeks, these patients continued their medication, although dosages will be dynamically adjusted based on treatment response. For example, if a patient’s condition stabilises, the sertraline dosage may be halved.

DBT-only group: The DBT intervention in this study focused on group skills training delivered by licensed psychotherapists who had received specialised training in DBT principles and application, with a minimum of (e.g., 2 years) of clinical experience in its delivery. The intervention adhered to the core principles outlined in established DBT treatment manuals, adapted for an adolescent outpatient group setting. Each group consisted of approximately 8 participants, and skills training sessions were conducted once per week, with each session lasting 90–120 minutes, for a total active treatment duration of 13 weeks. Following the active 13-week skills training, DBT-specific intervention was discontinued and an observation period continued until Week 24. Throughout the active treatment phase, therapists continuously monitored patients’ conditions, behaviours and skills application. The group skills training systematically covered the four core DBT modules: mindfulness, distress tolerance, emotional regulation and interpersonal effectiveness skills. Notably, whilst standard DBT often includes individual therapy and phone coaching, this specific program, as implemented and retrospectively analysed, centred on the group skills training component.

Combined pharmacotherapy and DBT (DBT+sertraline group): Patients received medication treatment and DBT simultaneously. DBT treatment continued until week 13, after which the DBT treatment was stopped; whereas sertraline continued to be administered until Week 24.

### Assessment of Treatment Efficacy

The primary metrics for assessing treatment efficacy include the Chinese versions of the HAMD and CERQ, both of which have been validated for reliability and validity in Chinese population samples and have been shown to demonstrate good psychometric properties [22, 23]. The HAMD was used to quantitatively assess the severity of depressive symptoms, whilst the CERQ analysed changes in emotional regulation, particularly covering 9 subscales encompassing positive and negative emotional regulation strategies.

### HAMD 

This scale comprises 24 items scored on a 5-point scale (0 = none, 1 = mild, 2 = moderate, 3 = severe, 4 = very severe), in which higher scores indicate more severe depression. Specifically, scores of <8 indicate no depressive symptoms, 8–20 mild depression, 20–34 moderate depression and ≥35 severe depression [24].

### CERQ

This questionnaire consists of 36 items, scored from 1 = never to 5 = always. It includes subscales such as Self-blame, Acceptance, Rumination, Positive refocusing, Refocus on planning, Positive Reappraisal, Putting into perspective, Catastrophising and Blaming others. Here, Positive refocusing, Refocus on planning, Positive Reappraisal and Putting into perspective are considered positive regulation strategies (20 items), whilst Self-blame, Rumination, Catastrophising and Blaming others are negative strategies (16 items). Higher scores on each scale indicate a greater tendency to use that particular cognitive-emotional regulation strategy when facing stressful events [25].

### Statistical Methods

All statistical analyses were performed using SPSS software (version 22.0; IBM Corp., Armonk, NY, USA). Data visualisation was conducted with GraphPad Prism (version 9.0; GraphPad Software LLC, San Diego, CA, USA). The normality of all continuous variables was initially assessed using the Shapiro-Wilk test. Normally distributed continuous variables are presented as mean ± standard deviation (SD). Between-group comparisons were analysed using two-way repeated measures ANOVA followed by Tukey’s post hoc test, with *p*-values adjusted via the Bonferroni method. For nonnormally distributed continuous variables, data are expressed as median (interquartile range, IQR) and analysed using the Mann-Whitney U test or Kruskal-Wallis test. Categorical variables are reported as frequencies and percentages, with comparisons performed using Chi-square tests. In a 2 × 2 table, Fisher’s exact test is used for between-group comparisons when any cell count is less than 5. Two-tailed *p*-values < 0.05 are considered statistically significant.

## Results

### Patient Information

This study included data from 148 adolescents, of whom 88 were diagnosed with depressive disorders and sought treatment at Lanzhou Petrochemical General Hospital from January 2023 to December 2023. The remaining 60 adolescents were healthy adolescents who neither exhibited symptoms of depression nor sought treatment for depressive disorders, thus serving as the control group. We collected data on all participants’ gender, age, residency status, only-child status, parents’ marital status, parents’ relationship, frequency of parental arguments, relationship with father, relationship with mother and school ranking (Table 1).

**Table 1.** **Comparison of basic demographic data between the control group and adolescents with depressive disorders**.

		Control	Adolescent depressive disorders	Test methods	Z/χ^2^	*p* value
Total		60	88			
Sex				Chi-square	8.20	0.004
	Female	26 (43.3)	59 (67.0)			
	Male	34 (56.7)	29 (33.0)			
Age				Mann–Whitney test	–2.26	0.041
	Median (P25, P75)	14.5 (14, 16)	16 (14, 17)			
Live site				Chi-square	37.87	<0.001
	Rural	32 (53.3)	7 (8.0)			
	Urban	28 (46.7)	81 (92.0)			
Religious belief				Fisher’s exact test	/	>0.999
	No	60 (100)	87 (98.9)			
	Yes	0 (0)	1 (1.1)			
Allergy history				Fisher’s exact test	/	>0.999
	No	58 (96.7)	84 (95.5)			
	Yes	2 (3.3)	4 (4.5)			
Only child				Chi-square	79.59	<0.001
	No	46 (76.7)	5 (5.7)			
	Yes	14 (23.3)	83 (94.3)			
Parental marital status				Chi-square	0.40	0.528
	Marriage	55 (91.7)	83 (94.3)			
	Divorce	5 (8.3)	5 (5.7)			
Parents’ relationship				Chi-square	3.62	0.164
	Bad	1 (1.7)	2 (2.3)			
	Fair	10 (16.7)	6 (6.8)			
	Good	49 (81.7)	80 (90.9)			
Parents argue				Fisher’s exact test	/	<0.001
	No	58 (96.7)	53 (60.2)			
	Yes	2 (3.3)	35 (39.8)			
Relationship with mother				Chi-square	3.80	0.150
	Bad	2 (3.3)	3 (3.4)			
	Fair	10 (16.7)	27 (30.7)			
	Good	48 (80)	58 (65.9)			
Relationship with father				Chi-square	11.68	0.003
	Bad	0 (0)	2 (2.3)			
	Fair	13 (21.7)	41 (46.6)			
	Good	47 (78.3)	45 (51.1)			
School rank				Chi-square	62.29	<0.001
	Fair	19 (31.7)	82 (93.2)			
	Good	41 (68.3)	6 (6.8)			

Significant differences were observed between adolescents with depression and the control group in terms of gender, age, residency status, only-child status, whether parents argued at home, relationship with fathers and school ranking (*p* < 0.05). Notably, a higher percentage of females was found in the depression group (*p* = 0.004), and the median age was slightly higher (*p* = 0.041). Adolescents from urban areas were more prevalent in the depressed group (*p* < 0.001). Furthermore, there was a significantly higher proportion of only children in the depression group (*p <*0.001). Families where parents often argued were more common in the homes of depressed adolescents; in contrast, nonargumentative families constituted the majority in the control group (*p* < 0.001). Relationship with the father had a more substantial impact on adolescent depression; in families with depression, the proportion maintaining a good relationship with the father was lower than in normal families (*p* = 0.003). Regarding school rankings, a higher percentage of patients attended regular schools, whilst a smaller percentage attended key schools (*p* < 0.001).

Patients with depression were divided into three treatment groups: 28 in the DBT group, 31 in the pharmacotherapy (sertraline) group and 29 in the combined treatment group (Table 2). At the beginning of the treatment (Week 0), there were no significant differences amongst all patients in terms of gender, age, residency status, only-child status, parents’ marital status, parents’ relationship, frequency of parental arguments, relationship with parents and school ranking (*p* > 0.05).

**Table 2.** **Basic demographic data of adolescents included in the treatment for depressive disorders**.

		Sertraline	DBT	DBT+ sertraline	Test method	H/χ^2^	*p* value
Total		31	28	29			
Sex					Chi-square	0.55	0.760
	Female	22 (71.0)	19 (67.9)	18 (62.1)			
	Male	9 (29.0)	9 (32.1)	11 (37.9)			
Age					Kruskal-Wallis test	2.82	0.748
	Median (P25, P75)	16 (14, 17)	15 (14, 16)	16 (14, 17)			
Live site					Chi-square	3.90	0.143
	Rural	3 (9.7)	0 (0)	4 (13.8)			
	Urban	28 (90.3)	28 (100)	25 (86.2)			
Religious belief					Chi-square	1.86	0.395
	No	30 (96.8)	28 (100)	29 (100)			
	Yes	1 (3.2)	0 (0)	0 (0)			
Allergy history					Chi-square	3.71	0.157
	No	30 (96.8)	28 (100)	26 (89.7)			
	Yes	1 (3.2)	0 (0)	3 (10.3)			
Only child					Chi-square	0.37	0.841
	No	2 (6.5)	1 (3.6)	2 (6.9)			
	Yes	29 (93.5)	27 (96.4)	27 (93.1)			
Parental marital status					Chi-square	2.90	0.223
	Marriage	29 (93.5)	28 (100)	26 (89.7)			
	Divorce	2 (6.5)	0 (0)	3 (10.3)			
Parents’ relationship					Chi-square	4.80	0.309
	Bad	2 (6.5)	0 (0)	0 (0)			
	Fair	2 (6.5)	1 (3.6)	3 (10.3)			
	Good	27 (87.0)	27 (96.4)	26 (89.7)			
Parents argue					Chi-square	4.74	0.093
	No	14 (45.2)	20 (71.4)	19 (65.5)			
	Yes	17 (54.8)	8 (28.6)	10 (34.5)			
Relationship with mother					Chi-square	3.21	0.523
	Bad	2 (6.5)	0 (0)	1 (3.4)			
	Fair	9 (29.0)	7 (25)	11 (37.9)			
	Good	20 (64.5)	21 (75)	17 (58.6)			
Relationship with father					Chi-square	4.88	0.300
	Bad	2 (6.5)	0 (0)	0 (0)			
	Fair	16 (51.6)	13 (46.4)	12 (41.4)			
	Good	13 (41.9)	15 (53.6)	17 (58.6)			
School rank					Chi-square	4.28	0.118
	Fair	29 (93.5)	28 (100)	25 (86.2)			
	Good	2 (6.5)	0 (0)	4 (13.8)			

Note: DBT, dialectical behaviour therapy.

### Impact of Treatment Methods on HAMD Scores

**Fig. 1.**
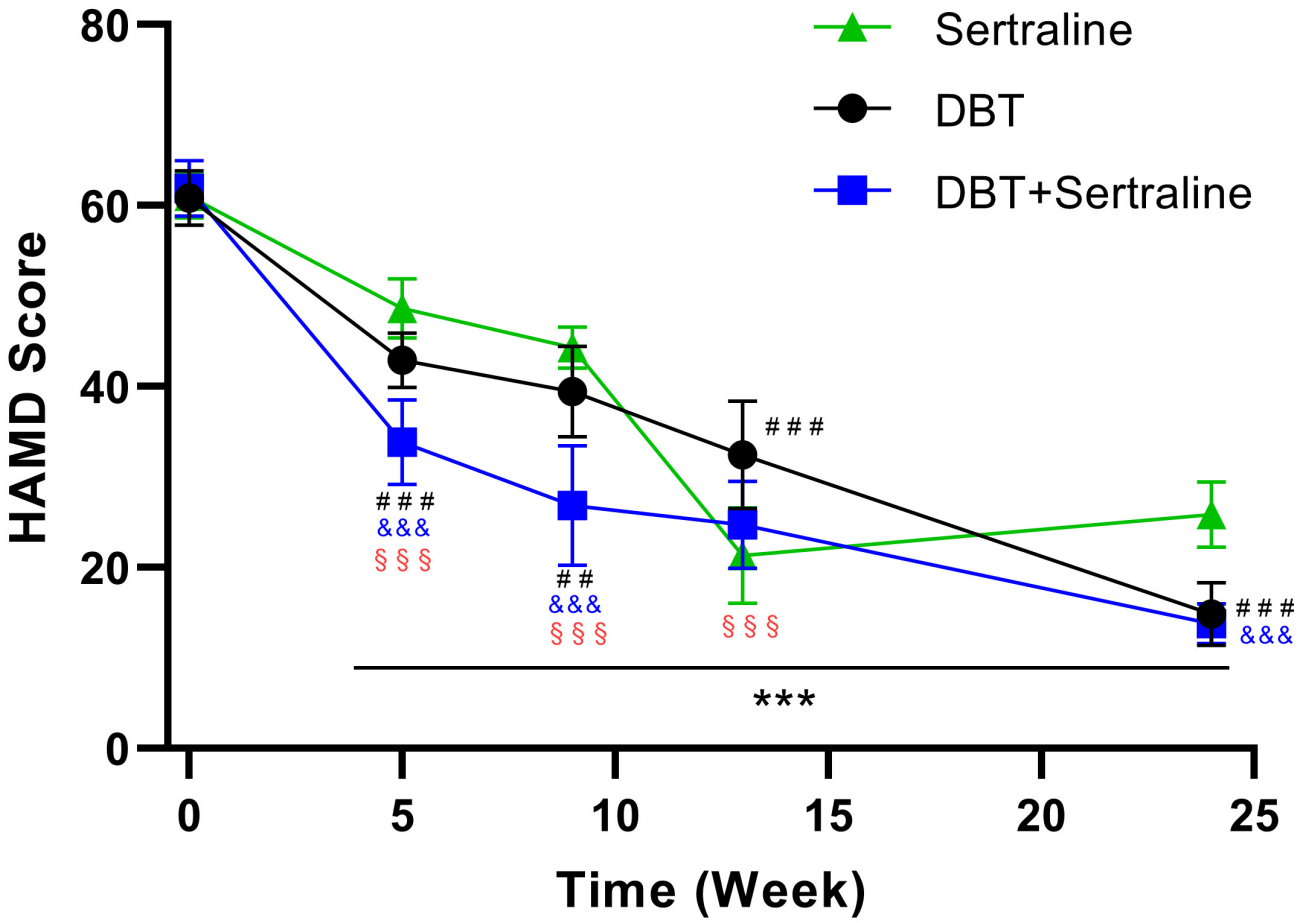
**Effects of different treatment methods on HAMD scores**. *** *p* < 0.001, indicates within-group comparisons of data at Weeks 5, 9, 13 and Week 21 versus Week 0. ^#⁢#^*p* < 0.01, ^#⁢#⁢#^*p* < 0.001, represent comparisons between DBT and sertraline at specific time points; ^&⁣&&^*p* < 0.001 denotes comparisons between DBT+sertraline and sertraline at specific time points; ^§⁢§⁢§^*p* < 0.001 indicates comparisons between DBT+sertraline and DBT at specific time points. HAMD, Hamilton Depression Rating Scale; DBT, dialectical behaviour therapy.

The results showed a significant reduction in HAMD scores across all treatment groups after six months of therapy (*p* < 0.001). In the first 9 weeks of treatment, the rate of reduction in the Sertraline-only group was significantly slower than in the DBT-only and DBT+sertraline treatment groups (Fig. 1 and Table 3). During the initial stages of treatment (Weeks 5 and 9), the DBT+sertraline group demonstrated the most rapid reduction in HAMD scores (Table 3, Fig. 1). By Week 13, all treatment groups showed substantial improvements from baseline, with the Sertraline-only group exhibiting the lowest mean HAMD score at this specific timepoint, followed by the DBT+sertraline group.

**Table 3.** **The impacts of different treatment modalities on HAMD scores over time**.

	DBT	Sertraline	DBT+sertraline	F	*p*	Partial Eta-squared (η^2^)
Week 0	60.8 ± 3.0	61.0 ± 2.5	61.9 ± 3.1	1.2	0.30	0.028
Week 5	42.9 ± 3.1	48.6 ± 3.3	33.9 ± 4.7	114.2	<0.001	0.729
Week 9	39.5 ± 5.0	44.3 ± 2.3	26.9 ± 6.6	93.0	<0.001	0.686
Week 13	32.5 ± 6.0	21.3 ± 5.3	24.7 ± 4.9	33.4	<0.001	0.440
Week 24	14.8 ± 3.5	25.9 ± 3.6	13.8 ± 2.2	126.7	<0.001	0.749
F	673.5	500.0	858.8			
*p*	<0.001	<0.001	<0.001			
Partial Eta-squared (η^2^)	0.973	0.965	0.976			

Note: DBT, dialectical behaviour therapy.

Notably, the DBT-only and DBT+sertraline groups showed a continuous decline in HAMD scores from Weeks 13–24. The Sertraline-only group showed a significant reduction in HAMD scores at Week 13 compared to Week 0 (*p* < 0.001), indicating long-term treatment effectiveness. However, by the 24th week, this group experienced a rebound in treatment effectiveness.

### Impact of Treatment Modalities on Cognitive-Emotional Regulation

Before (Week 0) and after treatment (Week 24), all participants completed the CERQ assessment (Table 4). The primary findings indicated that prior to treatment, scores for positive emotional regulation strategies were lower than those for negative emotional regulation strategies. After treatment, all three treatment methods significantly increased scores for positive emotional regulation strategies and significantly decreased scores for negative strategies.

**Table 4.** **Changes in cognitive-emotional regulation strategy scores before and after treatment**.

	Sertraline (N = 31)		DBT (N = 28)		DBT+ sertraline (N = 29)	
	Week 0	Week 24	Week 0	Week 24	Week 0	Week 24
Self-blame	13.2 ± 2.1	12.1 ± 1.5***	12.3 ± 1.4	10.1 ± 2.8***^,###^	12.5 ± 2.7	10.0 ± 2.4***^,###^
Acceptance	9.3 ± 1.2	11.8 ± 1.5***	9.4 ± 1.8	15.3 ± 3.5***^,###^	10.8 ± 2.6	18.9 ± 2.9***^,###,§§§^
Rumination	12.2 ± 2.3	10.9 ± 1.7^b)^	11.9 ± 2.4	10.3 ± 2.4^b)^	12.6 ± 2.4	11.4 ± 3.0^a)^
Positive refocusing	9.1 ± 1.8	11.8 ± 1.4***	9.4 ± 1.6	15.7 ± 3.9***^,###^	10.2 ± 1.6	17.8 ± 3.7***^,###,§^
Refocusing on planning	9.6 ± 1.9	11.4 ± 2.2*	9.0 ± 1.7	16.2 ± 2.8***^,###^	10.7 ± 1.6	18.9 ± 3.5***^,###,§§§^
Positive reappraisal	10.3 ± 2.0	13.1 ± 2.3***	8.9 ± 1.2	15.3 ± 2.4***^,##^	9.7 ± 2.1	18.6 ± 2.7***^,###,§§§^
Putting into perspective	9.2 ± 1.9	12.5 ± 2.1***	9.5 ± 2.1	15.0 ± 3.3***^,##^	10.3 ± 2.6	17.7 ± 2.9***^,###,§§§^
Catastrophising	12.5 ± 3.1	11.1 ± 2.6^a)^	13.1 ± 3.1	11.6 ± 1.5^c)^	11.8 ± 1.5	10.2 ± 2.4^c)^
Blaming others	11.9 ± 2.8	10.5 ± 1.5	12.4 ± 1.8	11.0 ± 1.6	13.9 ± 2.6^#⁢#^	11.8 ± 1.7**

Note: * *p* < 0.05, ** *p* < 0.01 and *** *p* < 0.001 indicate comparisons between Week 24 data and Week 0 within the same group; ^#⁢#^*p* < 0.01 and ^#⁢#⁢#^*p <*0.001 denote comparisons of the dialectical behaviour therapy (DBT) and DBT+sertraline groups versus the Sertraline group at the same time point; ^§^*p* < 0.05, ^§⁢§⁢§^*p* < 0.001 represent comparisons between the DBT+sertraline group and the DBT group at the same time point. Data were analysed using repeated-measures two-way ANOVA, followed by Tukey’s post hoc test, with Bonferroni correction for *p*-values. A *p*-value < 0.05 was considered statistically significant. ^a^, ^b^ and ^c^ represent the effect sizes (Cohen’s d values) for within-group comparisons between Week 24 and Week 0 data. Specifically, ^a^ indicates a small effect size (0.2–0.5); ^b^, a medium effect size (0.5–0.8) and ^c^, a large effect size (0.8–1.3).

Comparative analysis revealed that the DBT-only and DBT+sertraline groups achieved significantly greater improvements in these positive emotional scores compared to the Sertraline-only group at Week 24 (*p* < 0.001). Notably, the DBT+sertraline cohort showed statistically significant advantages over DBT-only in positive emotional scores at the Week 24 (*p* < 0.001). The most substantial improvement was observed in the Positive Reappraisal scores, which increased from 9.7 (Week 0) to 18.6 (Week 24) in the DBT+sertraline group. These findings indicate that, for the positive emotion scores, DBT+sertraline had the most significant increase, followed by the DBT group and, finally, the Sertraline group.

Regarding negative emotional control strategies, scores in Self-blame, Rumination, Catastrophising and Blaming Others all showed reductions. All three interventions showed significantly reduced scores in the Self-blame item compared to baseline measurements (*p* < 0.001). Whilst Rumination and Catastrophising scores showed numerical reductions across treatment groups, these changes did not reach statistical significance. The Blame others term demonstrated significant improvement only in the combination therapy group (*p* < 0.01 vs baseline). Furthermore, intergroup comparisons revealed that the DBT-only and DBT+sertraline groups achieved significantly greater reductions in Self-blame scores compared to the Sertraline-only group (*p* < 0.01).

## Discussion

Adolescent depressive disorders are increasingly recognised as a serious global public health issue. According to the WHO, depression is amongst the most common mental health challenges in adolescents, with a rising incidence over the past few decades [9]. The causes of adolescent depressive disorders are multifaceted, encompassing a variety of sociopsychological factors, as supported by existing literature and data from this study. Specifically, aspects such as the patient’s gender, age, place of registration, and whether they are an only child, can influence the manifestation of depressive symptoms [26, 27]. The present study reveals that female adolescents and those from urban areas generally face a higher risk of depression. The demographic trends observed in the current study are supported by a previous work [28]. However, divergences in the magnitude of effects observed in this study compared to that reported in prior literature may be attributed to the relatively small sample size and single-centre design, thus underscoring the need for future multicentre research with larger sample sizes. Family environment factors, such as the marital status of the parents, the quality of parental relationships, frequency of domestic arguments and individual relationships with each parent, also play crucial roles in influencing adolescent depressive symptoms. These findings suggest that a stable and supportive family environment may help mitigate the risk of depression in adolescents.

The current treatment modalities for adolescent depressive disorders primarily include psychotherapy, such as cognitive behaviour therapy (CBT), DBT and family therapy, along with pharmacotherapy, including SSRIs [29]. DBT is an evidence-based psychosocial therapeutic method that has shown efficacy in treating a range of psychological disorders, including depression, borderline personality disorder, substance abuse, anxiety disorders, severe depression and eating disorders [30].

In a study aimed at assessing the effectiveness of dialectical behaviour group therapy (DBGT) on stress, depression and cognitive-emotional regulation in mothers of students with intellectual disabilities, depression and stress scores significantly decreased after receiving DBGT treatment, while cognitive reappraisal and cognitive-emotional regulation scores significantly increased. The results indicate that DBGT had a positive impact on the psychological health of these mothers [31]. A clinical study implementing DBT for adolescents (DBT-A) to address suicidal and self-harming behaviours in adolescents found a significant reduction in nonsuicidal self-injuries during treatment [32]. Another study reported that DBT significantly reduced self-harming and suicidal ideation, decreased suicide attempts, alleviated depressive symptoms and lessened the frequency of self-injurious behaviours, thereby enhancing emotional regulation and social adaptability in a relatively short period [33]. A meta-analysis including 12 published studies similarly demonstrated significant reductions in self-harming behaviours and improvements in depressive symptoms following a course of DBT treatment in adolescents [34].

The aforementioned research findings are highly consistent with the results of the current study. By the end of this study (24 weeks), DBT-only treatment significantly reduced HAMD scores, decreased scores for negative emotional regulation strategies and increased scores for positive emotional regulation strategies. The mechanism by which DBT significantly improved depression is that it helped patients achieve balance between acceptance and change, enhanced their tolerance to painful situations and increased their self-efficacy, ultimately aiding patients in better managing their feelings, behaviours and relationships with others [35, 36].

Pharmacotherapy is also a primary treatment for childhood and adolescent depression. Current antidepressant medications include tricyclic and tetracyclic antidepressants, monoamine oxidase inhibitors, selective 5-HT reuptake inhibitors and 5-HT and norepinephrine reuptake inhibitors. However, as children and adolescents are still in the developmental phases, they may respond differently to antidepressants compared to adults [37].

Sertraline has been demonstrated in numerous studies to be effective for the treatment of depression in children and adolescents. Sertraline acts by blocking the reuptake of the neurotransmitter serotonin by neurons, thereby increasing the concentration of serotonin in the synaptic gap and enhancing neurotransmission to improve depressive symptoms [14]. In a study involving the treatment of adolescents aged 12–18 years with Major Depressive Disorder or Dysthymic Disorder using sertraline, results showed that it is safe, well-tolerated and significantly improved the clinical symptoms of depression [38]. A meta-analysis also showed that sertraline outperformed other antidepressants and can be considered a first-choice option for patients with severe depression [39]. Sertraline can also serve as an adjunctive therapy to other treatment modalities. For example, a study exploring the efficacy of repetitive transcranial magnetic stimulation combined with sertraline in treating severe depression in adolescents showed that using sertraline alone reduced HAMD-17 scores and combining it with repetitive transcranial magnetic stimulation could further reduce said scores [40]. Our findings are consistent with these results. In particular, our results showed that by the end of the study, there were significant reductions in HAMD scores and those for negative emotional regulation strategies, along with significant increases in scores for positive emotional regulation strategies before and after sertraline monotherapy.

However, it is worth noting that at Week 24, the HAMD scores in the Sertraline group showed a rebounding trend. The rebound in symptoms observed may be attributed to several factors. First, sertraline primarily alleviates depressive symptoms by modulating serotonin levels, but it does not address underlying cognitive-emotional regulation deficits. Sertraline does not enhance patients’ abilities to cope with stress, regulate emotions or achieve self-acceptance [41]. Thus, patients in this group may lack the coping skills necessary to manage stressors during the posttreatment phase, thereby increasing the risk of symptom relapse. In contrast, the impacts of the DBT group on patients are long-lasting, as it teaches them skills to handle difficulties and solve problems when encountering challenges.

Second, patients’ medication dosages were dynamically adjusted based on their conditions, and they continued to take sertraline after the conclusion of this study (24 weeks). Based on the relevant literature, the potential reasons for sertraline-induced rebound lie in its pharmacokinetic properties and neuroadaptation mechanisms. With a moderate half-life of 22–36 hours, sertraline is rapidly metabolised, causing a sharp decline in plasma concentration after administration. In turn, this may trigger the rebound overactivation of neurotransmitter systems like serotoninergic hypersensitivity, leading to symptom recurrence or exacerbation. Meanwhile, long-term use of sertraline downregulates the density of serotonin transporters, inducing adaptive changes in the central nervous system, which may trigger severe symptom fluctuations (e.g., rebound depression and anxiety) [42].

Third, it is also possible that over time, patients may develop tolerance to sertraline or their adherence to daily medication may decrease, leading to insufficient or irregular dosing—both of which could contribute to rebound effects. However, it should be noted that whilst this phenomenon has been clinically observed, the specific and clearer mechanisms or causes have not been fully elucidated. As such, these findings not only underscore the limitations of pharmacotherapy as a standalone treatment for adolescent depression but also highlight the importance of integrating psychotherapy, such as DBT, into pharmacotherapy to achieve more sustainable long-term outcomes.

When comparing the Sertraline-only group with the DBT group, the latter showed better outcomes, with quicker reductions in HAMD scores at initial stage and higher scores for positive emotional regulation strategies. Our findings align with previous results indicating that patients who exhibit higher levels of Positive Reappraisal and lower levels of negative coping (e.g., Self-blame and Rumination) after treatment show greater improvements in depressive symptoms [43]. This finding underscores the importance of focusing not only on alleviating symptoms but also on enhancing emotional regulation capabilities during the treatment of adolescent depression, which is crucial for patients’ long-term recovery and social function restoration.

Our study also assessed the efficacy of combined sertraline and DBT treatment for adolescent depressive disorders. We found that the combined treatment outstripped the benefits of DBT or sertraline alone, with the fastest reductions in HAMD scores at the initial treatment stage. Additionally, scores for positive emotional regulation strategies in the combined treatment group were significantly higher than those in the DBT-only or sertraline-only groups. Specifically, the most significant improvement was observed in the Positive Reappraisal scores, which may play a role in preventing mental health issues by enhancing social functioning. Therefore, the marked increase in Positive Reappraisal scores holds substantial clinical significance for patients with depression. On the one hand, improved reappraisal skills can directly reduce depressive and anxiety symptoms by disrupting cycles of rumination and catastrophising. On the other hand, by fostering Positive Reappraisal, the combined DBT and sertraline intervention can build psychological resilience, reducing patients’ vulnerability to stressors after treatment [43, 44]. In summary, the improvement in Positive Reappraisal scores has a tangible, positive impact on functional recovery, quality of life and the long-term maintenance of mental health.

Several potential mechanisms could underpin this synergistic effect. Pharmacotherapy with sertraline may more rapidly alleviate core neurovegetative and affective symptoms of depression, such as anergia or profound dysphoria, thereby enhancing adolescents’ cognitive and emotional capacity to actively engage with and benefit from the skills-based components of DBT [13]. Conversely, DBT offers practical skills for managing emotional crises, improving interpersonal effectiveness and tolerating distress, which can address psychosocial stressors that medication alone may not resolve. These learned skills may also empower adolescents to better manage medication side effects, improve treatment adherence and navigate residual symptoms, leading to a more comprehensive and sustained clinical improvement [45]. Such a dual approach, which targets the neurobiological pathways and maladaptive coping patterns, is theorised to yield more robust outcomes in complex psychiatric conditions [19]. This notion suggests that in treating adolescent depression, both single-drug treatment and combined treatment strategies have their advantages and limitations and that long-term treatment effects may be influenced by multiple factors. Our finding is consistent with those reported in previous studies. For instance, in a meta-analysis comparing the effects of antidepressant medication, psychotherapy and combined treatment, the combined treatment exhibited an obvious advantage over individual therapeutic approaches [20].

However, there are also studies that have arrived at different conclusions. In a study evaluating the effects of CBT, sertraline and combined CBT and sertraline in treating adolescent depression, results showed that all three treatment methods significantly improved depression symptoms. Notably, the study revealed that the combined therapy was not more advantageous than the individual treatments [46]. In fact, a meta-analysis evaluating a large sample of patients found that compared to either pharmacotherapy or psychotherapy alone, the effects of combined treatment were smaller [47].

Another meta-analysis that examined the effects of treating chronic depression using a combination of medication and psychotherapy found that the combined treatment had a statistically insignificant effect on directly related depressive outcomes [48]. These findings indicated that combination therapy appeared to demonstrate no significant advantage over monotherapy, which contrasts with our results showing that the combined approach significantly outperformed standalone treatments in efficacy. Furthermore, these findings [48] indicated that combination therapy appeared to demonstrate no significant advantage over monotherapy, which contrasts with our results showing that the combined approach significantly outperformed standalone treatments in terms of efficacy.

We infer that this inconsistency may be caused by many reasons. First, the meta-analyses that reported smaller or statistically insignificant effects for combined treatment primarily focused on adult populations with depression or specifically examined chronic depression. The adolescent demographic in our study may respond differently to interventions due to this population group’s unique developmental considerations and illness presentations. Second, our study population exhibited exceptionally high baseline HAMD scores, signifying very severe depression. It is conceivable that the synergistic benefits of combined pharmacotherapy and intensive psychotherapy like DBT are more pronounced and clinically necessary in individuals at the higher end of the severity spectrum. In contrast, meta-analyses often include a broader range of patient severities, and the additive value of combination therapy might be less evident in milder or more heterogeneous samples. Third, these differing research findings may also be attributed to variations in age distribution, family factors, treatment duration, patient compliance with the different treatment modalities and variations in therapist skills. Therefore, large-scale clinical studies are still required to systematically evaluate the effects of drug treatment, DBT treatment and combined treatments.

Notably, the baseline HAMD scores of all three groups in this study were exceptionally high. Whilst DBT alone showed significant therapeutic effects, the severity of the initial symptoms highlights the challenges of managing such cases with DBT in isolation. The results underscore the importance of using multimodal approaches, as the combined DBT+sertraline group demonstrated the most substantial improvements, suggesting that pharmacotherapy may play a critical role in stabilising severe symptoms whilst DBT addresses emotional regulation and coping skills.

Studies have shown that antidepressant treatment may be associated with higher side effects, such as headaches and rashes [39]. Therefore, the efficacy and adverse reactions of antidepressant drugs in treating childhood and adolescent depression must be confirmed by large-sample analyses. Furthermore, future research should explore how sociopsychological factors affect adolescent depressive symptoms through specific biopsychosocial mechanisms and optimise treatment strategies based on these findings to provide more precise clinical guidance.

This study has several limitations. First, our assessment of treatment outcomes relied on clinician-rated (HAMD) and self-reported (CERQ) measures. Whilst these are well-validated instruments for assessing depressive symptom severity and cognitive emotion regulation strategies, respectively, the absence of objective biological measures, such as functional magnetic resonance imaging (fMRI) or biochemical markers, limits our ability to draw definitive conclusions about the underlying neurobiological mechanisms of action for either DBT or sertraline. Consequently, any discussion of mechanisms in the present study, such as how these interventions might impact brain function or neurochemistry leading to symptomatic or cognitive changes, is inferential and based on the observed changes in these clinical and cognitive scores. As such, future research incorporating these objective measures would be invaluable for elucidating these mechanisms and further validating the observed therapeutic effects.

Second, as this was a single-centre study conducted in a specific hospital setting, the findings may not be fully representative of adolescent populations in other geographical locations or healthcare contexts. This restriction limits the generalisability of our findings and may introduce selection bias related to referral patterns or patient characteristics typical of this centre. Therefore, future investigations should adopt a multicentre design to enhance the generalisability of the results.

Third, the sample size of this study was relatively small, and the patient group exhibited specific demographic features (e.g., predominantly urban and a high proportion of only children). This feature may limit the statistical power to detect more subtle effects and the applicability of the findings to more sociodemographically diverse adolescent populations. Thus, subsequent studies should aim to expand the sample size and recruit from more varied populations. Fourth, future studies should prioritise recruiting healthy control participants with matched general characteristics (e.g., sex, age, residential background and only-child status) to minimise confounding effects. Fifth, this study did not systematically measure patient compliance with the different treatment modalities or variations in therapists’ skill levels in providing DBT.

Furthermore, whilst clinical monitoring for medication side effects was a component of standard care for patients receiving sertraline, the retrospective design and the nature of routine clinical record-keeping in this study did not permit a systematic quantification of specific side effect incidence rates per treatment group with the rigor required for comparative research analysis. These unmeasured factors (i.e., encompassing adherence levels, detailed side-effect profiles per group and pharmacokinetic differences) could potentially influence treatment outcomes, including the observed rebound phenomenon in the Sertraline-only group, thus representing a significant limitation in fully interpreting these findings. Future prospective studies would benefit from incorporating standardised measures to systematically collect and report on adherence, pharmacokinetics and side effects. These factors could also potentially influence treatment outcomes and contribute to variability in results both within our study and across the wider literature. Therefore, their exploration is warranted in future research to better understand the nuances of treatment efficacy and the conditions under which combined therapies may offer optimal benefits.

## Conclusion

This study compared the therapeutic effects of DBT alone, sertraline alone and the combination of DBT and sertraline on adolescents with depressive disorders using the HAMD and the CERQ. The results demonstrated that all three treatment modalities (DBT only, Sertraline-only and DBT+sertraline) significantly reduced HAMD scores. However, the Sertraline-only treatment group experienced a rebound in symptoms at the end of the study. The DBT+sertraline treatment was the most effective, followed by the DBT-only group. The CERQ scores indicated that the DBT alone and DBT+sertraline groups significantly improved their positive emotional regulation strategies and reduced their negative emotional regulation strategies.

Furthermore, this study shows that the DBT alone and DBT+sertraline groups not only effectively alleviate symptoms of depression but also significantly improve adolescents’ emotional regulation capabilities. In fact, their therapeutic effects are superior to those of the sertraline-only treatment group. However, when selecting a treatment plan, it is essential to consider patients’ specific conditions, treatment responses and individual differences to achieve the best therapeutic outcomes.

## Availability of Data and Materials

The datasets used or analysed during the current study are available from the corresponding authors on reasonable request.
